# Study on the Properties and Structure of Rotationally Moulded Linear Low-Density Polyethylene Filled with Quartz Flour

**DOI:** 10.3390/ma15062154

**Published:** 2022-03-15

**Authors:** Karolina Głogowska, Przemysław Pączkowski, Bronisław Samujło

**Affiliations:** 1 Department of Technology and Polymer Processing, Faculty of Mechanical Engineering, Lublin University of Technology, Nadbystrzycka 36, 20-618 Lublin, Poland; b.samujlo@pollub.pl; 2 Department of Polymer Chemistry, Institute of Chemical Sciences, Faculty of Chemistry, Maria Curie-Skłodowska University in Lublin, Gliniana 33, 20-614 Lublin, Poland; przemyslaw.paczkowski@umcs.pl

**Keywords:** rotational moulding, quartz flour, mineral filler, compatibility agent, low-density polyethylene

## Abstract

The objective of this study is to determine selected properties of thin-walled rotationally moulded composite parts. Linear low-density polyethylene (LLDPE) filled with quartz flour (QF, 5–35 wt.%) was tested. High-density polyethylene functionalized with maleic anhydride (HDPE-g-MA) was used as a compatibility agent. Polymer samples were prepared with and without the compatibility agent (2 wt.% in relation to the QF content). The study investigated the effect of QF content and HDPE-g-MA on the properties of rotationally moulded parts, including their melt flow rate (MFR), thermal properties (DSC and TGA), thermomechanical properties (VST), mechanical and physical properties, microstructure, and geometry. Results showed that the properties of LLDPE/QF with HDPE-g-MA were significantly higher than those of LLDPE/QF without HDPE-g-MA. It was also found that the compatibility agent improved the composite material’s thermal stability. This improvement was attributed to interactions occurring between the composite material components due to the use of the compatibility agent. In addition to that, microscopic examination demonstrated that the use of HDPE-g-MA improved miscibility of the composite material components. The composite samples containing HDPE-g-MA had better surface geometry.

## 1. Introduction

The use of organic and inorganic natural waste materials in the production of polymer products is a recent trend in polymer processing. Filled plastics can be processed using machines such as extruders [1], injection moulding machines [2,3] and rotational moulding machines [4].

Rotational moulding is a high-temperature processing method in which the molten polymer is spread over the walls of the mould cavity by the centrifugal force [5,6]. In this process, the mould performs either rotary or planetary motion about the main and auxiliary axes that are perpendicular to each other or it rotates about its own axis while performing swinging motion [7]. Rotational moulding is predominantly used to produce hollow parts, open or closed, with a capacity of up to ten thousand litres [8]. It is possible to accurately reproduce the mould cavity shape [9]. A considerable advantage of rotational moulding is that this process is conducted under atmospheric pressure, which makes it cost-effective compared to high-pressure processes [10].

**Figure 1:**
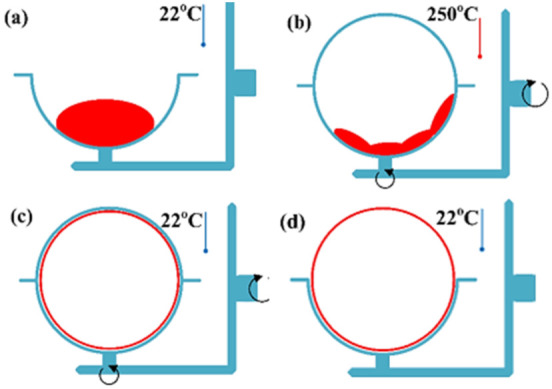
Four stages in rotational moulding: (**a**) loading, (**b**) heating, (**c**) cooling and (**d**) unloading.

Rotational moulding is a cyclic process consisting of four stages: loading, heating, cooling and unloading (Figure 1) [9,10,11,12,13].

**Figure 2:**
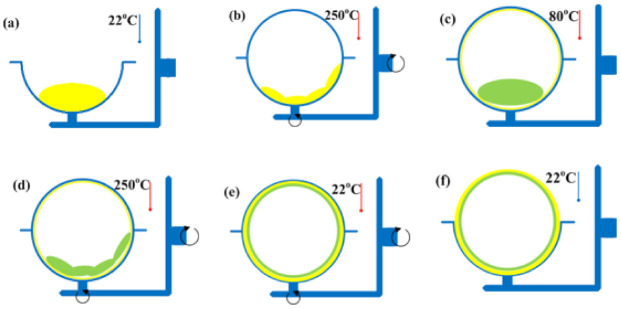
Stages in rotational moulding of double-layer products: (**a**) first loading, (**b**) first heating, (**c**) second loading, e.g., of a blowing agent, (**d**) second heating, (**e**) cooling, (**f**) unloading.

Thanks to recent developments in rotational moulding, this technique can now be used to produce multilayer and porous products [4,14,15]. Such products are manufactured by sequentially adding various polymers and auxiliaries (Figure 2) [16,17]. After melting, spreading and cooling the first polymer material, another polymer or solid auxiliary (e.g., powder or microgranulate) is poured into the mould cavity. The mould is rotated about the main and auxiliary axes. The second layer of the material is formed on the inside of the first layer by adhering particles [18]. Auxiliary agents are added to change physical and chemical properties of a material and thus obtain mouldings with required functional properties.

The growing interest in rotational moulding and the widespread use of this technique in many industries leads to the production of materials with improved quality and properties. This, in turn, results in a greater availability of such materials, as well as the development of new cost-effective materials. The use of rotational moulding to produce composite materials has become very popular [5,19,20]. The growing interest in environment protection has resulted in numerous studies on advanced materials made of natural raw materials, such as polymer-matrix composites. The production of composites that are based on renewable and natural waste materials has become of interest to many researchers in materials engineering and polymer processing. Compared to widely popular mineral fillers and glass and carbon fibers, the use of natural fillers and fibers can be more beneficial due to their reduced price, low density and environmental friendliness (rapid biodegradation).

Key aspects that must be taken into consideration in composite material design include the following: polymer and filler selection, phase interaction at the polymer–filler interface, filler dispersion in the polymer, processing method, as well as filler orientation in the polymer. Polymer composite processing methods depend on the polymer type and filler form (powder, fiber, flakes). To achieve the desired properties of a polymer composite, proper bonding between the polymer and the filler must be ensured during the production process.

**Figure 3:**
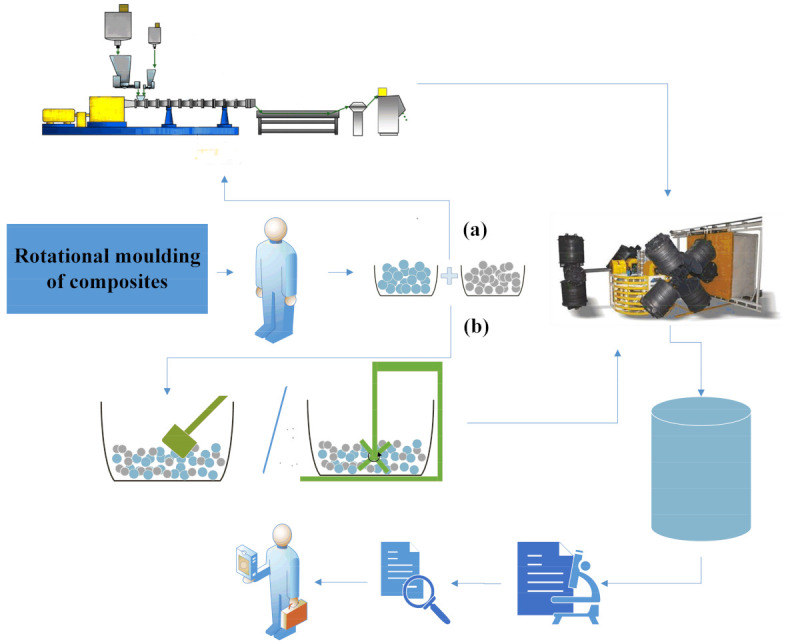
Scheme illustrating the fabrication of composites by rotational moulding: (**a**) mixing filler with polymer matrix by extrusion (materials—extrusion—granulation—rotational moulding—product—research–sales), (**b**) without extrusion (materials—manual or mechanical mixing—rotational moulding—product—research–sales).

One of the most important operations in the production of composites is mixing. Thin-walled composite mouldings can be produced in two ways (Figure 3) [21]. The first method consists of first mixing the filler with the polymer matrix by extrusion and then granulating or grinding the extrudate (Figure 3a). It should be remembered that the use of additional processing to obtain thin-walled mouldings may lead to their higher production costs and structural changes. The other method involves no extrusion (Figure 3b). Before rotational moulding, the filled polymer is mixed either manually or mechanically and then fed into the mould cavity.

In a study by Höfler et al. [22], the batch material (polyethylene—PE/halloysite nanotubes—HNT) was prepared by three different methods: it was dry mixed manually and with a high-speed mixer, and it was compounded using a twin-screw extruder. The extrudate was granulated with a granulator and milled to the required particle size. It was observed that dry mixing of the material with a high-speed mixer reduced the agglomeration of HNT particles in the polymer matrix, when compared to manual dry mixing. It was also found that the use of mixing, granulating and milling before rotational moulding led to improved surface quality of thin-walled mouldings, as well as reduced filler particle agglomeration and pinholes, which was also observed for higher HNT contents. The use of twin-screw extruder mixing resulted in improved mechanical properties of all PE/HNT samples. In light of the results obtained by Höfler [22], Hejna et al. [23] decided to omit melt mixing. Linear low-density polyethylene (LLDPE) with wheat bran was dry mixed with a high-speed mixer. The rotational speed of the mixer was 2000 rpm and the materials were mixed for 5 min. The composite compound was dried in a laboratory dryer at 70 °C for 24 h. Fletes et al. [4] fabricated compounds of agave fibres (CBA) and linear medium density polyethylene (LMDPE) by dry mixing with a high-speed mixer at 1750 rpm for 5 min. Before rotational moulding, the material was dried for 12 h at 60 °C. In [24], polyethylene (PE) and polylactide (PLA) samples were dried at 60 °C for 24 h, and a buckwheat husk (BH) filler was dried at 100 °C for 48 h. It was found that good mixing and appropriate preparation of the batch materials led to increased homogeneity of the composite, which—in turn—affected physical, chemical and functional properties of thin-walled mouldings.

Fillers are used not only to improve functional properties of polymer materials but also to reduce their production costs. In this study, a mineral filler in the form of quartz flour (QF) was added to linear low-density polyethylene (LLDPE). Two types of samples were rotationally moulded: with and without a compatibility agent. The use of silica as a reinforcing filler may cause various problems due to significant differences in the polarity of silica and polyethylene. The silica surface may aggregate or single particles may be covered with polar and chemically active silanol groups (-Si-OH). The maleic anhydride groups grafted onto polyethylene lead to enhanced PE polarity and thus complex interaction between the anhydride and hydroxyl groups on the silica surface. All samples were prepared in the same way. They were thoroughly examined in terms of mass melt flow rate, thermal, mechanical, thermomechanical and physical properties, as well as microstructure and surface roughness.

## 2. Materials and Methods

The study on the production and properties of LLDPE/QF composites included preliminary research that was related to the following: optimization of technological parameters of their production in a rotational moulding machine (selection of rotational speeds about the main and auxiliary axes, furnace chamber temperature, heating and cooling times); design of the composite compound (share and type of powder filler, polymer material and compatibility agent); and design of the production method (preparation of raw materials, adding/mixing order of batch materials).

### 2.1. Materials

The literature review [5] on the use of plastics for rotational moulding processes showed that the most suitable plastic for the present study was linear low-density polyethylene (LLDPE) that comes in powder form, i.e., DOWLEX^®^ 2629UE (LLDPE). This polymer material is manufactured by the Dow Chemical Company (Schkopau, Germany). This plastic is characterized by high efficiency and good processing. It has many applications; it is used for blowing extrusion, injection moulding, extrusion moulding for sections, and rotational moulding. Basic properties of the tested polymer are listed in Table 1.

A natural mineral filler in the form of quartz flour (QF) with the trade name MK.075/001 was used in the study. The filler is manufactured by Strzeblowska Kopalnia Surowców Mineralnych Sp. z o.o. (Wrocław, Poland). This quartz flour is made of natural quartz sand ground to dust. According to the manufacturer’s data, the main component of QF is silicon oxide (min. 99.0%); it also contains aluminium oxide (max. 0.50%), iron oxide (max. 0.05%) and titanium oxide (max. 0.05%). Silicon oxide (SiO_2_) is hydrophilic but can be chemically modified to make it hydrophobic. To this end, silanes are used as non-ionic surfactants with reactive groups binding the surface of the mineral filler to the polymer matrix.

Quartz flour particles are characterized by an irregular shape. The standard density of this mineral filler is 2650 kg m^−3^ and its bulk density is 756 kg m^−3^. The filler is light grey in colour. Quartz flour is used as a filler, modifier of polyester resins, paints and varnishes; it is also used for the production of paints, grouts, adhesives, as well as in the rubber and construction industry. QF can be added into the polymer matrix either in an unmodified form or via organophilization with a silane compatibility agent.

**Figure 4:**
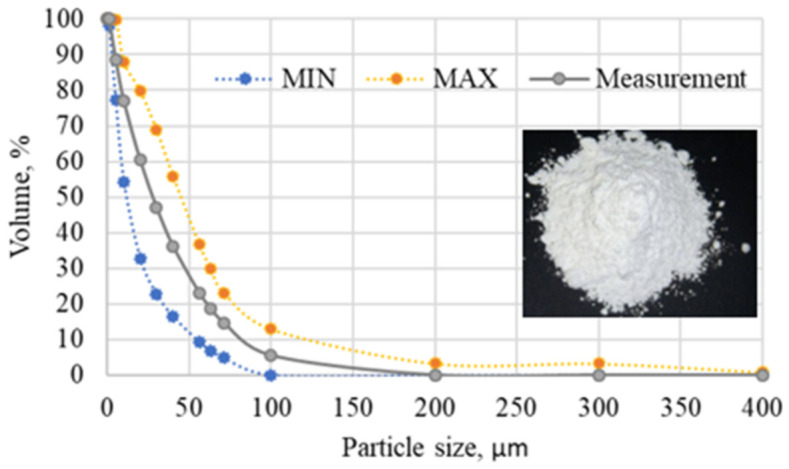
Distribution of particle volume fraction as a function of quartz flour grain size.

To determine the QF particle size distribution, the manufacturer used a laser granulometer from Malvern Instruments (Warsaw, Poland). The distribution of particle volume fraction as a function of quartz flour grain size is shown in Figure 4.

High-density polyethylene (HDPE) functionalized with maleic anhydride was used as a compatibility agent. It comes under the trade name SCONA TPPE 1212 PAHD (HDPE-g-MAH) in powder form. It is manufactured by BYK—Chemise GmbH (Wesel, Germany). This compatibility agent has a melt flow rate of 0.5–2 g/10 min (190 °C, 2.16 kg). Following the manufacturer’s recommendations, 2% of the compatibility agent was added, based upon the mass content of the mineral filler (QF).

### 2.2. Rotational Moulding of Polymer Composites

Prior to the rotational moulding process, the walls of the mould cavity were cleansed with the MOLD PREP CLEANER SOLVENT TR-905 mould cleaning fluid; after that, the MULTI-PULL TR-910 sealing primer was applied. The last step was to apply the release agent MULTI—PULL LIQUID SEMI—PERMANENT RELEASE TR-930. The abovementioned chemicals are manufactured by TR Mold Release (South Gate, CA, USA). The layers were applied according to the manufacturer’s instructions.

The conditioning process of linear low-density polyethylene filled with quartz flour consisted of drying it in a laboratory drier for 24 h at 70 °C. Prior to rotational moulding, appropriate quantities of the batch material were mechanically dry-mixed in a planetary mixer. A loose mixture was obtained; it was poured directly into the mould cavity of the rotational moulding machine. The total amount of the material used in the production of each part was 460 g.

**Figure 5:**
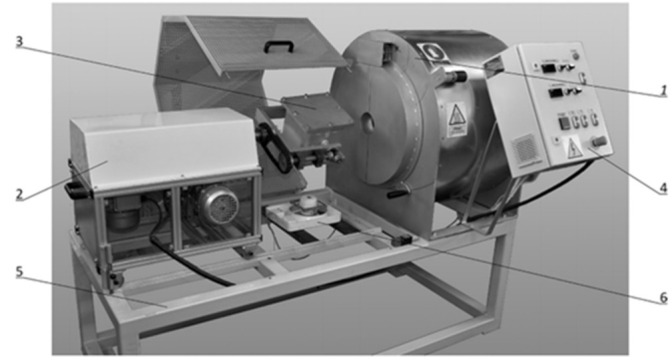
Laboratory machine for rotational moulding: 1—heating system, 2—rotational system, 3—mould, 4—control and regulation system, 5—base, 6—fan.

The rotational moulding process was performed on a single-arm spindle-type machine constructed by the Institute for Engineering of Polymer Materials and Dyes (Toruń, Poland). The rotational moulding machine was equipped with a cube-shaped steel mould with the mould cavity dimensions of 200 mm × 200 mm × 200 mm (Figure 5).

**Figure 6:**
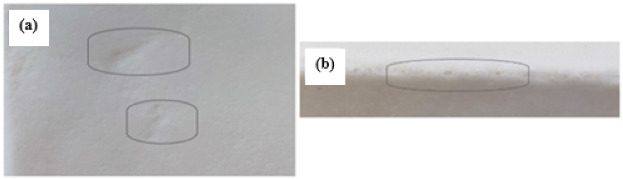
Photographs of surface defects in (**a**) wall and (**b**) corner of a thin-walled moulding containing 15 wt.% of quartz flour (QF).

Following the addition of the appropriate amount of the mixture, the mould was closed and put into rotation, and then it was pushed into the furnace chamber. The rotation speed on the main axis was 10 rpm, while the rotation speed on the auxiliary axis was 5 rpm. The temperature in the furnace chamber was 250 °C. The heating and cooling of the material in the mould took 20 min. The mould was cooled by forced air flow. Thin-walled mouldings with a 15% quartz flour content had surface defects and material losses in their walls (Figure 6a) and corners (Figure 6b). Therefore, it was decided that the mould cooling method should be changed and that the cooling time should be extended. Successive composite mouldings were air-cooled for 40 min. Thanks to the application of the different cooling method and cooling time, no surface defects were observed on the composite mouldings (Figure 6). The corners of the mouldings were correct. All thin-walled mouldings were produced under the same technological conditions.

Mouldings containing 5 to 35 wt.% of quartz flour were produced along with analogous systems compatibilized with 2% of high-density polyethylene functionalized with maleic anhydride. It was not justified to use higher contents of the mineral filler. Following the removal of the composite mouldings containing 35 wt.% of the mineral filler, QF deposit could be observed on the walls of the mould cavity and on the mould covers.

### 2.3. Measurements

The assessment of the properties of produced thin-walled mouldings involved the following.

A load plastometer from INSTRON (Turin, Italy) was used for melt flow rate testing. The test stand was provided with additional equipment and a precision balance from RADWAG (Radom, Poland). Measurements of the mass melt flow rate (MFR) of the polymer mixture were conducted in compliance with the ISO 1133 standard [25]. The test conditions were: 190 °C/2.16 kg.

Thermal properties of LLDPE materials were determined by differential scanning calorimetry (DSC) and thermogravimetric analysis (TGA). Calorimetric measurements were made using a DSC calorimeter from NETZSCH (Selb, Germany) according to the heat–cool–heat procedure. The first heating cycle from −150 °C to 180 °C, the cooling cycle from 180 °C to −150 °C, and the second heating cycle from −150 °C to 180 °C were performed at a heating/cooling rate of 10 °C min^−1^ under an argon atmosphere (gas flow rate of 30 mL min^−1^). The samples were tested in aluminium crucibles with a pierced lid. As a reference, an empty crucible was used. The glass transition temperature ($T_{g}$) of the samples was determined at the temperature of thermal curve inflection point. The crystallization temperature ($T_{c}$) and the melting point ($T_{m}$) of the samples were obtained as the maximum thermic peak. The melting enthalpy ($\Delta H_{m}$) and the degree of crystallinity ($X_{c}$) were determined. The degree of crystallinity of pure LLDPE and its polymer composites ($X_{c}$) was calculated using the following Equation (1) [26].

(1) $$X_{c}=\frac{\Delta H_{m}}{\omega \times \Delta H_{m}^{{}^{\circ}}}\times 100$$

where: $\Delta H_{m}$ is the melting enthalpy of the sample, $\Delta H_{m}^{{}^{\circ}}$ is the melting enthalpy for 100% crystalline polyethylene, and ***ω*** is the weight fraction of polymer matrix.

Relative crystallinity was calculated assuming that $\Delta H_{m}^{{}^{\circ}}$ of 100% crystalline LLDPE is 293 J g^−1^ [27].

Thermogravimetric analysis (TGA) was carried out using a thermal analyser from NETZSCH (Selb, Germany) at temperatures from 30 to 900 °C under a synthetic air atmosphere (gas flow rate of 20 mL min^−^^1^) with a heating rate of 10 °C min^−1^. Samples weighing about 10 mg were tested in an open Al_2_O_3_ crucibles. The mass loss temperatures ($T_{1\%}$, $T_{5\%}$, $T_{10\%}$, $\;T_{50\%}$), peak maximum decomposition temperatures ($T_{max}$), and residual mass (***RM***) were determined. The thermal analysis measuring instruments were supported by the NETZSCH Proteus software, version 6.0.0 (Selb, Germany).

Thermomechanical tests were complemented with Vicat softening temperature (VST) measurements. A device from INSTRON (Turin, Italy) equipped with three workstations was used for the thermomechanical tests. The Ceast View 6.12A software was used to control the analysed processes, as well as to report and export results. The Vicat softening temperature (VST) was measured with a load of 10 N and a heating rate of 50 °C h^−1^. Measurements were carried out in an oil bath in compliance with the ISO 306 standard [28]. To ensure correct realization of the softening point tests, two samples were stacked one on top of the other to obtain a measuring height of 4 mm.

The static tensile test of mechanical properties was carried out on a Zwick Roel testing machine (Ulm, Germany), in accordance with ISO 527-1: 2012 [29]. In the test, the traverse speed was 5 mm min^−1^, while the test speed of Young’s modulus at tensile stress was 1 mm min^−1^. The initial tensile force was 0.1 MPa. The above values of Young’s modulus and tensile strength were based on the average of at least seven samples.

Hardness measurements were made by the Shore D method. A digital Shore hardness tester from Bareiss Prüfgerätebau GmbH (Oberdischingen, Germany) was used. A head provided with a stylus indenter was used. Shore hardness measurements were made in accordance with the applicable ISO 868: 2003 standard [30]. The test load was set to 50 N. At least 7 measurements were made for each batch of the samples.

The immersion method was used to determine normal density. The test was performed in compliance with the ISO 1183-1 A standard [31].

A high-resolution digital microscope from Keyence (Mechelen, Belgium) was used for microstructure examination. The microscope consists of a motorized XYZ stage and a set of lenses enabling image magnification up to 2500×. The microscope had a number of adjustments made to its controls so that samples could be examined at a full depth of field, at various angles. This microscope also makes it possible to construct 3D profiles of samples and perform a roughness evaluation. High dynamic range (HDR) imaging enables a high colour gradation. Selected samples were subjected to microscopic examination that was performed under identical magnification and light conditions.

The investigation of surface geometric structure was performed for four samples from each type of thin-walled moulding. Tests were carried out on a device for measuring contour, roughness and 3D topography from Hommel-Etamic (Jena, Germany). The following roughness parameters were measured: *Ra*, *Rz*, *Rmax*, *Rq* and *RSm*. Measurements were made in compliance with ISO 11562 [32]. According to this standard, the selected profile roughness parameters are defined as follows: *Ra*—mean roughness|: the arithmetic average of the absolute values of the roughness profile ordinates; *Rz*—maximum height of the profile; *Rmax*—maximum roughness depth; *Rq*—root mean square deviation of the roughness profile; *RSm*—mean spacing of the profile elements. The TURBO WAVE V7.55 program was used to operate the device and perform measurements. The TKU300 probe tip was used in the tests. The measuring range was 400 µm. The mapping distance was set at 4.80 mm. The experiments were conducted at a speed of 0.80 mm s^−1^.

## 3. Results and Discussion

### 3.1. Melt Flow Rate (MFR)

**Figure 7:**
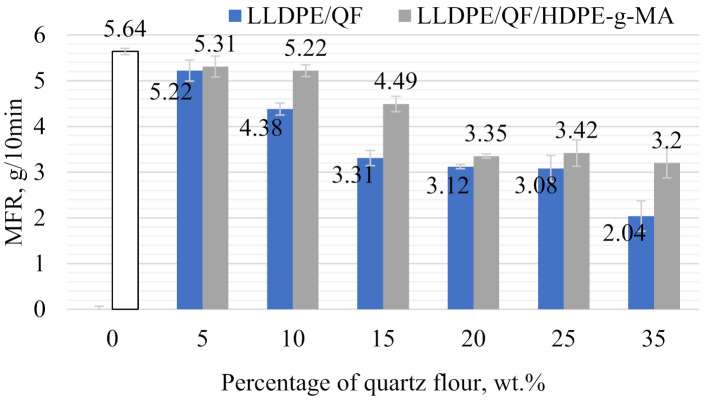
Melt flow rate of LLDPE/QF and LLDPE/QF/HDPE-g-MA.

A relationship between melt flow rate (MFR) and filler content and compatibility agent addition is shown in Figure 7. An analysis of the mass flow rate data demonstrates that the best results were obtained for the samples with a 5% mineral filler content, with and without the compatibility agent. For LLDPE/5%QF and LLDPE/5% QF/HDPE-g-MA, the mass melt flow rate is 5.22 g/10 min and 5.31 g/min, which amounts to, respectively, 7.44% and 5.85% decrease compared to the reference sample (unfilled). The lowest MFR was obtained for the samples containing 35% of the mineral filler, with and without HDPE-g-MA. For the LLDPE/5%QF and LLDPE/5%QF/HDPE-g-MA samples, the mass flow rate is 2.04 g/10 min and 3.2 g/10 min, which means that it decreased, respectively, to 63.82% and 43.26% of the value obtained in rotational moulding of the unfilled material. The results indicate a significant deterioration of the processability index of the composite samples compared to pure LLDPE. It is worth noting that the presence of HDPE-g-MA in the polymer compound improves its processing properties, when compared to those of the LLDPE/QF sample.

One of key processing properties of polymeric materials is melt viscosity. This parameter depends on the average molecular weight of the polymer, as well as on the type and content of fillers [33,34]. Melt flow rate is an indirect measure of the viscosity of polymer materials. The higher the melt viscosity of the polymer/polymer compound, the lower the melt flow rate is. A decrease in MFR with increasing the filler content is associated with increased viscosity of the filled material, as well as with an increase in its density (Figure 12) caused by increasing the amount of quartz flour particles in the matrix of linear low-density polyethylene. This phenomenon is well-known and often described in the literature [35,36]. A probable cause of the reduction in MFR is the ability of filler particles to agglomerate, which results in a distorted flow of the polymer material in the platometer die flow channels. QF particles may also increase friction of the polymer material against the scraps of the plastometer flow channel. The addition of the compatibility agent may lead to reducing the occurrence of the abovementioned phenomena. It is probable that HDPE-g-MA reduces the coefficient of friction between the polymeric material, the mineral filler, and the walls of the plastometer flow channel.

When analysing the processing properties of the rotationally moulded polymer composite samples, it should be remembered that they can only be useful for initial selection and description of the tested materials. Processability indices are determined under shear conditions that differ significantly from the conditions for plastics processing by rotational moulding.

### 3.2. Thermal Behaviour—DSC Analysis

**Figure 8:**
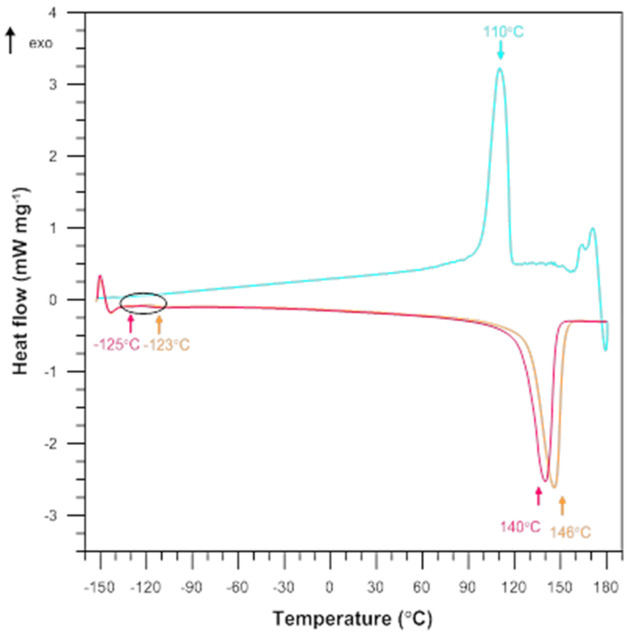
DSC curves for the pure LLDPE.

**Figure 9:**
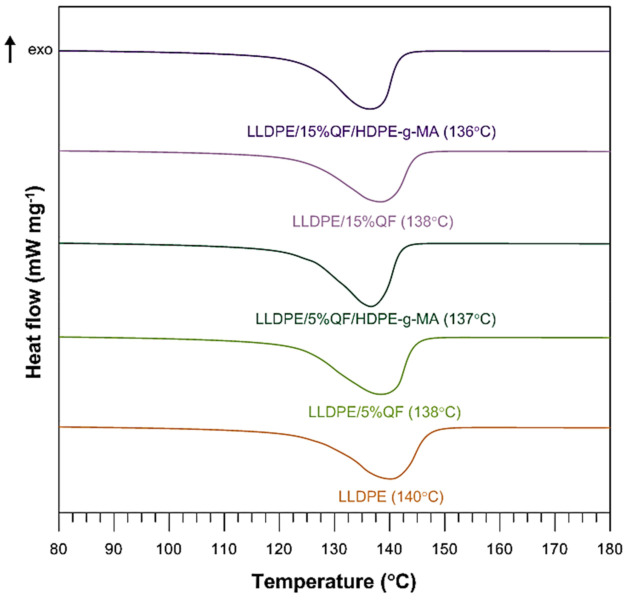
DSC 2nd heating cycle curves for LLDPE, LLDPE/QF and LLDPE/QF/HDPE-g-MA.

Calorimetric measurements were performed to determine the effect of silica on the differences in thermal behaviour and crystalline structure of LLDPE samples. Figure 8 and Figure 9 show the DSC heating and cooling thermograms obtained for the pure LLDPE and the silica-filled samples. The melting point of the samples ($T_{m}$) and the melting enthalpy ($\Delta H_{m}$) for the composite samples were obtained from the heating curves (first and second cycle). The crystallization temperature ($T_{c}$) was determined from the cooling curve. The degree of crystallinity of pure LLDPE and its polymer composites ($X_{c}$) was calculated using Equation (1).

Important DSC characteristic data obtained for pure LLDPE and modified samples are listed in Table 2.

An analysis of the DSC curves obtained under argon atmosphere reveals the presence of a few thermal events, both exothermic and endothermic. For the pure LLDPE sample, the endothermic peak in the first and the second heating cycle is assigned to the melting point ($T_{m}$) of 146 °C and 140 °C, respectively, while the glass transition temperature ($T_{g}$) is −123 °C and −125 °C, respectively. The exothermic peak corresponding to the crystallization temperature ($T_{c}$) that occurred during cooling is equal to 110 °C. With increasing the quartz flour content in the composite, the melting temperature slightly decreases by 3–4 degree compared to the pure LLDPE sample. The glass transition temperature decreases by 2–3 degree. The opposite can be observed for the crystallization temperature; increasing the filler content, the temperature increases from 110 to 112 °C. The degree of crystallinity of the composite samples increases with increasing the quartz flour content from 0 to 5%; after that, it decreases with increasing the filler content from 5 to 35%.

This can be explained by the fact the addition of quartz flour to LLDPE causes a reduction in the size of crystals and thus a slight decrease in melting point and increased crystallinity. It may also be related to the ability of silica particles to limit the macromolecular mobility and reduce the available space occupied by the polymer chains. In other words, the silica prevents LLDPE crystals from growing due to the small distances between the particles [37].

The silica-filled samples also include LLDPE functionalized with maleic acid anhydride (HDPE-g-MA). It can be observed that the changes are very small, only by 1–2 degrees, and hence, it is extremely difficult to clearly interpret the effect of this additive. During the cooling process $T_{c}$ is lower than that obtained for the samples without HDPE-g-MA and even almost the same as that obtained for pure LLDPE. The effect of grafting on the melting behaviour is also very limited.

The melting enthalpy of the composite samples increases with increasing the filler content from 0 to 5%; it decreases with increasing the silica content from 5 to 35%. The same relationship can be observed for the samples containing HDPE-g-MA; however, the values are much lower compared to the samples without the compatibility agent and even significantly lower than those obtained for pure LLDPE.

### 3.3. Thermogravimetric Analysis (TGA)

**Figure 10:**
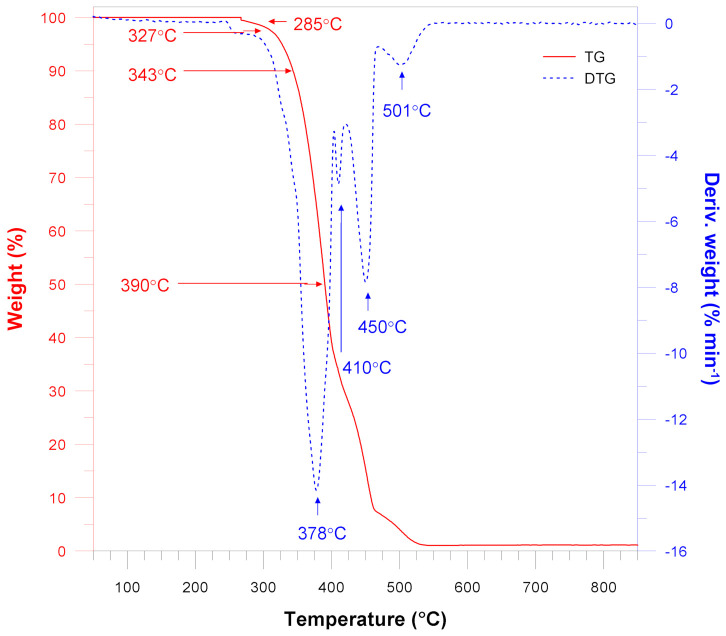
Thermal decomposition of the pure LLDPE.

**Figure 11:**
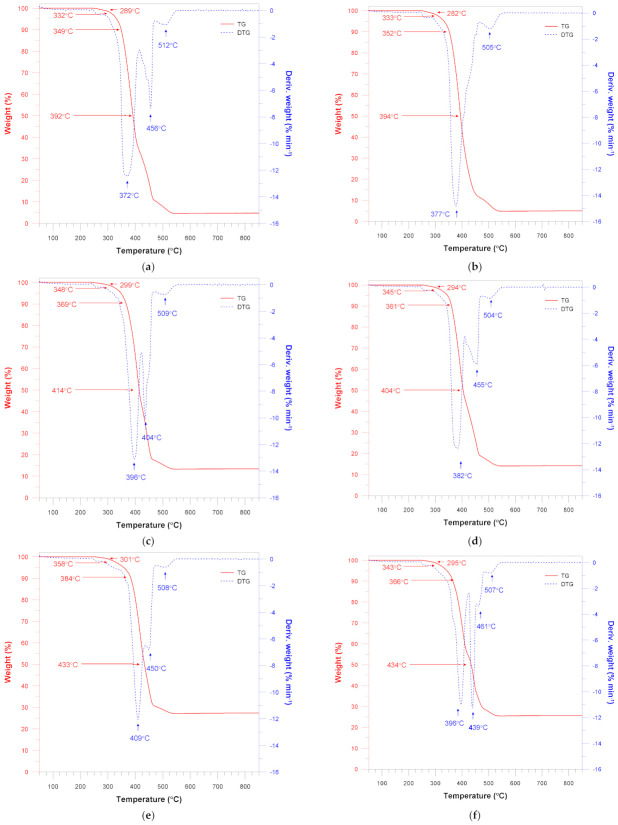
Thermal decomposition of the LLDPE/QF materials: (**a**) LLDPE/5%QF; (**b**) LLDPE/5QF/HDPE-g-MA; (**c**) LLDPE/15%QF; (**d**) LLDPE/15QF/HDPE-g-MA; (**e**) LLDPE/35%QF; (**f**) LLDPE/35QF/HDPE-g-MA.

TG and DTG thermal stability analyses of the pure LLDPE and silica-filled materials under oxidative atmosphere were performed (Figure 10 and Figure 11). The TG and DTG curves demonstrate that the pure LLDPE sample exhibits a few degradation steps with the decomposition maximum at 378 °C, 410 °C, 450 °C and 501 °C.

According to the DTG curves for all samples, the initial changes occur at about 250–260 °C, whereas the main decomposition process takes place above this temperature range and continues to about 600 °C. The DTG curves are almost similar to those obtained for pure LLDPE, but for the materials filled with quartz flour the peaks merge into one broad peak.

Nevertheless, it can be observed that increasing filler content results in higher characteristic temperatures. The decomposition temperature ($T_{1\%}$) of pure LLDPE is reached at 285 °C and it gradually increases to 301 °C with increasing the quartz flour content. A similar trend can be observed for the temperature at the mass loss 5%, 10% and 50%.

Similar observations regarding filler content can be made for the samples of LLDPE/QF containing HDPE-g-MA. However, their characteristic temperatures ($T_{1\%}$) are about 5–7 degrees lower than those of the unfilled samples.

According to thermogravimetric analysis, TG and DTG curves show, that all characterized materials were thermally stable up to about 280–290 °C.

Characteristic temperatures that were estimated based on the abovementioned curves are listed in Table 3. The addition of the malleated coupling agents leads to improved thermal stability of LLDPE. The increased adhesion between the polyethylene matrix and the SiO_2_ particles can be interpreted to be a result of intermolecular interactions taking place between the polar groups of maleic anhydrides and silanol groups on the silica surface. These interactions are responsible for reducing the size of silica aggregates by decreasing the filler–filler interaction and increasing the filler–polymer interaction instead [38].

As previously mentioned, the thermal stability increase may result from enhanced interaction between the polymer matrix and the silica particles, which stabilizes the entire system due to the limitations of thermal motions of the HDPE chains.

Thermogravimetric analysis results also show changes in residual mass. The pure LLDPE sample shows the presence of a small amount of residual mass amounting to 1.12%. This can be explained by the fact that the residual mass can come from other additives or ingredients that were added to the plastic during the manufacturing process. As expected, the increase in the quartz flour content from 0 to 35% causes an increase in the residual mass from 1.12 to 27.40%.

### 3.4. Vicat Softening Temperature (VST)

Results showing the effect of quartz flour content and compatibility agent addition on Vicat softening temperature (VST) are given in Table 4. An analysis of the results reveals minimal changes in the softening point. The VST of the unfilled polymer is 126.36 °C. The highest VST of 127.05 °C is obtained for the samples containing 10 wt.% of the filler and the compatibility agent. The lowest Vicat softening temperature equal to 122.91 °C is obtained for the samples with a 20% QF content without the compatibility agent. In the analysed cases, it is difficult to determine the impact of the mineral filler on VST due to the fact that the percentage values of the temperature changes do not exceed 2.59% of the initial value.

Based on the obtained test results it is difficult to establish a relationship between mineral filler content and VST. The behaviour pattern of VST is very unstable. The results of VST tests for rotationally moulded parts reported in the literature indicate a similar decrease in this parameter (by 1–3%) [23,34,39]. Usually, the decreased VST is caused by significant porosity of the tested material and poor dispersion of a filler in the polymer matrix.

It is worth noting that the samples with the compatibility agent are characterized by a higher VST than the LLDPE/QF samples. This may result from the fact that the compatibility agent improves the dispersion of the mineral filler in the polymer matrix.

### 3.5. Physical and Mechanical Properties

**Figure 12:**
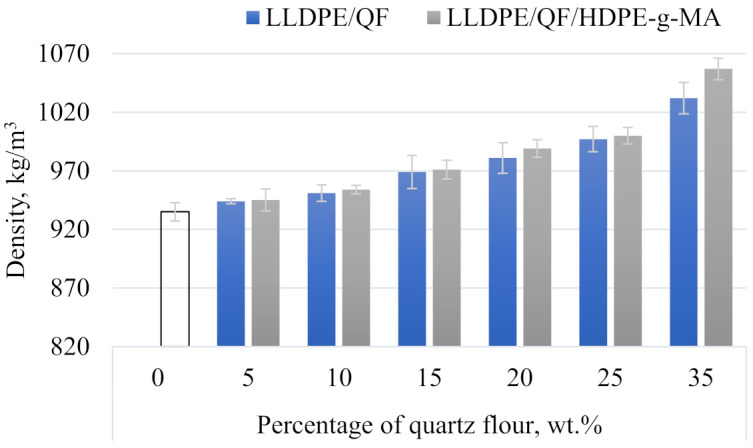
Normal density of LLDPE/QF and LLDPE/QF/HDPE-g-MA.

Figure 12 illustrates the relationship between mineral filler content and compatibility agent addition and the normal density of the samples. The addition of the highest tested content of the mineral filler leads to an increase in density from 935 kg m^−3^ to 1032 kg m^−3^ for LLDPE/QF and to 1057 kg m^−3^ for LLDPE/QF/HDPE-g-MA, i.e., the initial density increased by 10.37% and 13.04%, respectively. The observed changes in density result from the fact that the density of the tested filler is three times higher than that of linear low-density polyethylene. The samples with the compatibility agent are characterized by higher density in the entire tested range of this variable factor. The LLDPE/QF/HDPE-g-MA samples are probably less porous.

**Figure 13:**
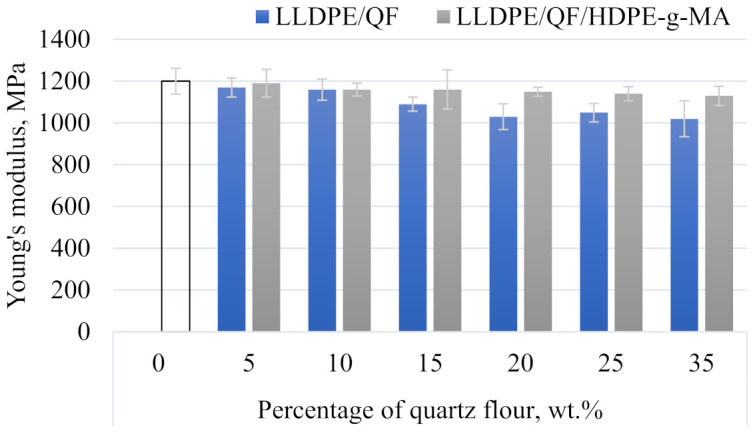
Young’s modulus of LLDPE/QF and LLDPE/QF/HDPE-g-MA.

Selected strength properties (Young’s modulus, tensile strength *σ_m_* and relative elongation at maximum tensile stress *ε_m_*) of both pure linear low-density polyethylene and modified samples are given in Figure 13 and Table 5. The results of the static tensile test demonstrate that the mineral filler content in the polymer and the addition of the compatibility agent are of significant importance.

An analysis of Young’s modulus (Figure 13) shows that this parameter significantly decreases in all modified samples compared to the unmodified linear low-density polyethylene. The addition of 35 wt.% of the mineral filler to the LLDPE matrix reduces the Young’s modulus from 1200 MPa to 1020 MPa, which amounts to 15% of the initial value. The lowest value of Young’s modulus can also be observed for the samples with the compatibility agent containing 35 wt.% of QF—Young’s modulus is equal to 1130 MPa, which amounts to a 5.83% decrease compared to the pure LLDPE sample.

The addition of 35 wt.% of the mineral filler causes a significant reduction in the tensile strength of linear low-density polyethylene. Compared to the unfilled sample, the reduction in this property amounts to 66.79% for the composite sample without the compatibility agent. As for the samples with the compatibility agent, a smaller decrease in tensile strength can be observed for the samples with a high filler content, this decrease amounts to 56.66% compared to the pure polymer sample.

The results show that relative elongation at maximum tensile stress decreases with increasing the filler content in the samples with and without HDPE-g-MA alike. The addition of 35 wt.% of the filler reduces the relative elongation at maximum tensile stress by 80% compared to the reference sample (unfilled). A smaller decrease for the same filler content can be observed for the samples with the compatibility agent, this decrease amounting to 53% compared to the pure LLDPE sample.

The decrease in strength properties is directly related to increased porosity of the composite samples and insufficient strength of interfacial interactions between the polymer and the filler [21,23]. Porosity leads to a reduced stress transfer capacity during the static tensile test and a reduction in the actual cross-sectional area of the tested material [40]. It also causes discontinuities in the structure of the filled material, which results in a significant deterioration of its mechanical properties [41]. The reduction in tensile strength can be attributed to the differences in the size of quartz flour particles and the filler distribution in the polymer matrix [42].

It can be assumed that the addition of the compatibility agent (i.e., high-density polyethylene functionalized with maleic anhydride) results in better dispersion of filler particles in the polymeric matrix and enhanced interfacial interactions, i.e., the adhesion between LLDPE and QF. As a result, the strength properties of the filled samples are higher than those of the samples without the compatibility agent.

The mechanical properties of composite materials have become lower than those of pure polymers; nevertheless, this fact does not exclude them from being used in the production of low-demanding parts. The porous structure of composite parts leads to reduced material density, which may be advantageous in some industrial applications such as the production of automotive parts.

**Figure 14:**
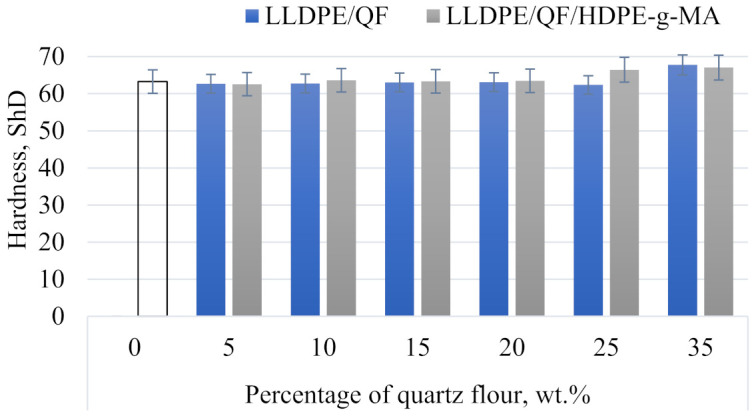
Shore hardness of LLDPE/QF and LLDPE/QF/HDPE-g-MA.

Hardness results are presented in Figure 14 as a relationship between Shore D hardness and different mass contents of the mineral filler. The hardness of the unfilled polymer material is 63.25°ShD. The highest hardness of 66.77°ShD was achieved by the samples containing 35 wt.% of the filler and the compatibility agent, which corresponds to a 5.56% increase compared to the unfilled material. The lowest hardness of 63.05°ShD was obtained for the samples containing 15 wt.% quartz flour without the compatibility agent.

### 3.6. Microscopic Structure

**Figure 15:**
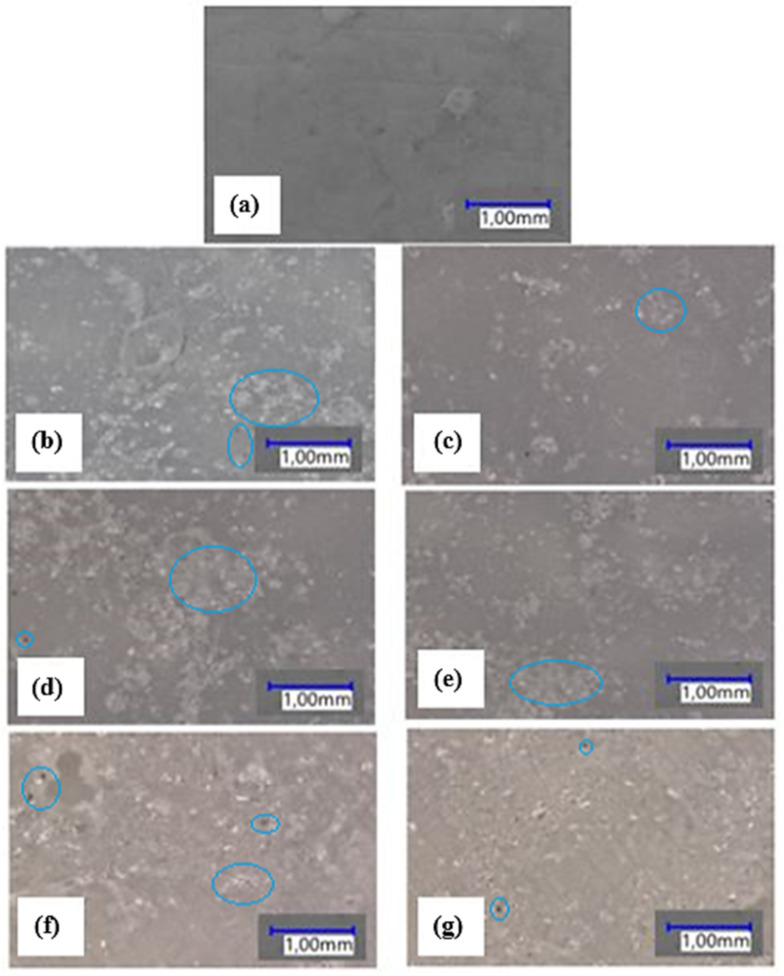
Micrographs of linear low-density polyethylene and composite samples: (**a**) pure LLDPE, (**b**) LLDPE/5%QF, (**c**) LLDPE/15%QF, (**d**) LLDPE/35%QF, (**e**) LLDPE/5%QF/HDPE-g-MA, (**f**) LLDPE/15% QF/HDPE-g-MA, (**g**) LLDPE/35%QF/HDPE-g-MA (examples of agglomerates and pores are marked in blue).

The microstructure of the analysed thin-walled mouldings is shown in Figure 15. A comparison of the micrographic images reveals that the QF-filled samples containing the compatibility agent are characterized by a uniform dispersion of QF in the polymer matrix. In contrast, the samples without the compatibility agent show the presence of agglomerates of the filler.

It can be observed that the composite samples contain pores in their structure; the pores were formed during processing. The unfilled linear low-density polyethylene has a homogeneous structure. The presence of porosity in the rotationally moulded composite samples may result from the size of filler and polymer particles and the reduced melt flow rate (Figure 7) [5]. The increased porosity indicates that the material requires longer heating in the furnace chamber or that it is necessary to use a material with a higher MFR [40,43,44].

In the samples containing HDPE-g-MA fewer pores can be observed, which suggests that the compatibility agent leads to a reduced coefficient of friction between the polymer/filler particles, and thus causes increased densification in the thin-walled composite mouldings.

### 3.7. Surface Roughness

**Figure 16:**
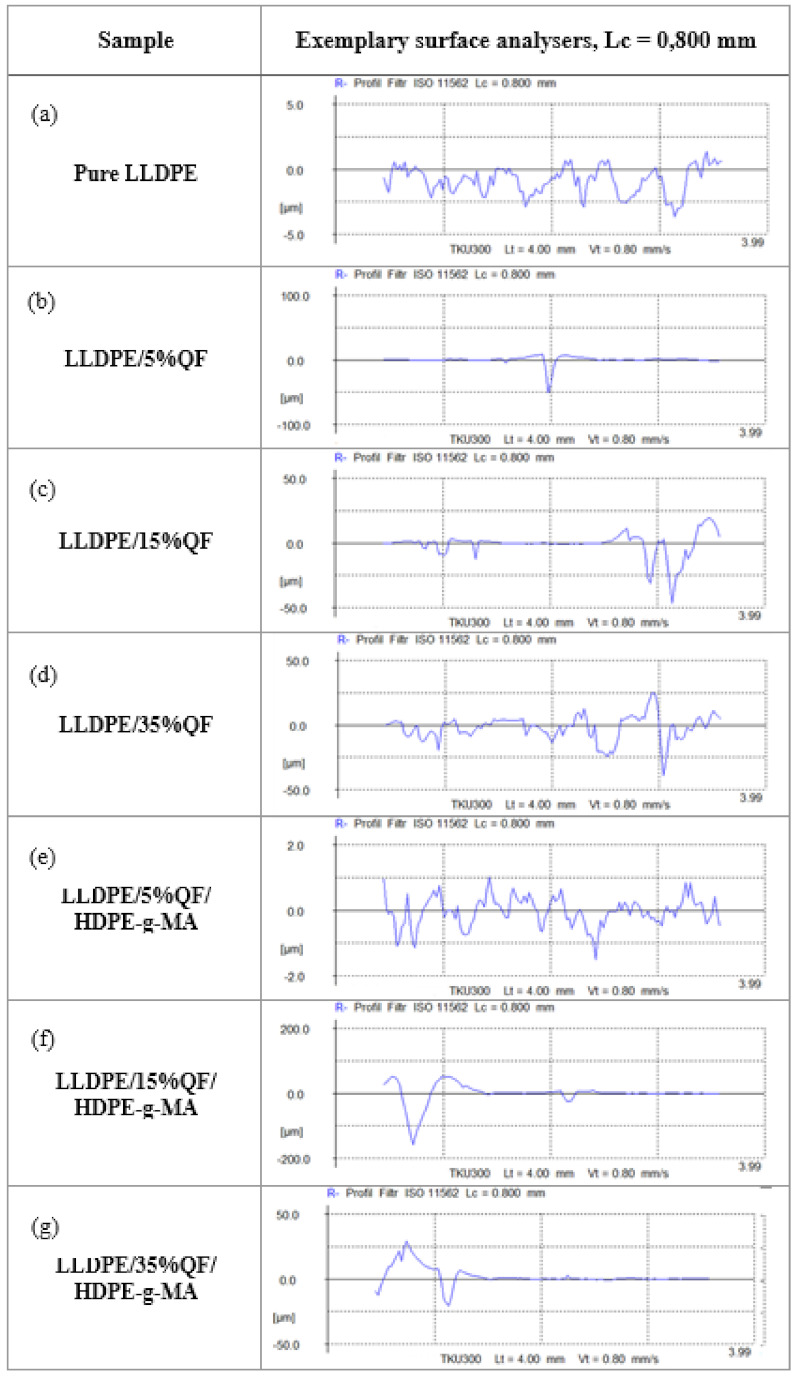
Profilograms of linear low-density polyethylene and composite samples: (**a**) pure LLDPE, (**b**) LLDPE/5%QF, (**c**) LLDPE/15%QF, (**d**) LLDPE/35%QF, (**e**) LLDPE/5%QF/HDPE-g-MA, (**f**) LLDPE/15%QF/HDPE-g-MA, (**g**) LLDPE/35%QF/HDPE-g-MA.

Results of selected surface roughness parameters of the tested thin-walled moulded parts are given in Table 6. The objective of surface roughness measurements was to determine some parameters of surface geometrical structure of the samples. Figure 16 shows the examples of profilograms obtained for the samples of pure LLDPE, LLDPE/QF (5%, 15% and 35%) and LLDPE/QF/HDPE-g-MA (5%, 15% and 35%).

The surface roughness results demonstrate that all tested parameters: *Ra*, *Rz*, *Rmax*, *Rq* and *RSm* increased their values compared to the unfilled polymer. The surfaces of the LLDPE/35%QF and LLDPE/35%QF/HDPE-g-MA samples show the highest values of *Rmax*. An analysis of the surface roughness results given in Figure 16 reveals that the compatibility agent has a positive effect on all tested parameters. The addition of HDPE-g-MA to the polymer compound results in improved geometric structure of the surface. Apart from the increased mineral filler content, another reason for the higher surface roughness may be the so-called “orange peel” (OP) effect, which was described in a study by E. Soos Takacs et al. [45]

The term “orange peel” describes a surface defect that is characterized by irregular grooves and pits, resembling the topography of an orange peel. The presence of the described surface defect is undesired not only for aesthetic reasons, but also because of its potential negative impact on the functional properties of thin-walled mouldings, e.g., their cleaning and mechanical properties. According to E. Soos Takacs et al. [45], increased heating time and temperature lead to reduced crystallinity and surface roughness.

The investigation into the effect of particle size on the formation of OP showed that small particles cause smaller OP. A comparison of the effect of the mixing method on the OP formation shows that dry mixing induces the formation of a rough orange peel. The results demonstrate that the difference in density of the polymer compound components has a greater impact on the formation of OP than the difference in MFR.

## 4. Conclusions

This study investigated the effect of adding different contents of a mineral filler in the form of quartz flour to the matrix of linear low-density polyethylene on selected properties of obtained composite samples. Two types of samples were rotationally moulded: with and without compatibility agent.

The results clearly demonstrate that the addition of different contents of the mineral filler to LLDPE has a negative or negligible effect on the strength, physical and structural properties of the samples and leads to increased surface roughness. A comparison of the samples with and without the compatibility agent has revealed that the addition of HDPE-g-MA led to increased Young’s modulus, tensile strength, relative elongation at maximum tensile stress, hardness and mass flow rate. The microstructure of quartz flour with the compatibility agent was characterized by a uniform dispersion of QF in the polymer matrix. The addition of HDPE-g-MA to the polymer compound resulted in reduced surface roughness.

The authors have also observed that the compatibility agent improved the thermal stability of the material, and they attribute this improvement to the interactions occurring between the composite components when using maleic anhydride-functionalized polyethylene. The results of Vicat softening temperature do not indicate that increased contents of the mineral filler and compatibility agent had any effect on this parameter.

Further studies on LLDPE/QF/HDPE-g-MA composites should focus on the assessment of changes in processing, structural, mechanical, physical and thermal properties of composites subjected to accelerated aging tests. It is also worth investigating how changing the mixing method will affect the tested parameters. Composites filled with quartz flour are an interesting topic for further scientific and research work. When selecting filler type and content, one must take into consideration various factors and parameters from disciplines such as chemistry, physical chemistry of polymers, mechanics, rheology, materials engineering, and even processing machinery design. With a properly selected filler content, the properties of a given composite material can be shaped as desired. It is thus important to conduct research on novel composite materials that contain new, previously unused fillers.

## Figures and Tables

**Table 1:** Basic properties of the polymer used in the tests.

Property	Value	Units
Density	935	kg m^−^^3^
Melt Flow Index (190 °C/2.16 kg)	4	g/10 min
Vicat Softening Point A120	119	°C
Deflection Temperature Under Load HDT B	65	°C
Melting Point	124	°C
Crystallisation Point	110	°C
Hardness, Shore D	57	°Sh
Tensile Yield Stress	17.5	MPa

**Table 2:** Calorimetric measurement characteristic data for LLDPE/QF materials.

Sample	Heat				Cool		Heat			
	1st Cycle						2nd Cycle			
	$T_{g}$ (°C)	$T_{m}$ (°C)	$\Delta H_{m}$ (J g^−1^)	$X_{c\;}$ (%)	$T_{c}$ (°C)	$\Delta H_{c}$ (J g^−1^)	$T_{g}$ (°C)	$T_{m\;}$ (°C)	$\Delta H_{m}$ (J g^−1^)	$X_{c}$ (%)
Pure LLDPE	−123	146	225	76.79	110	201	−125	140	213	72.70
LLDPE/5%QF	−126	145	243	87.30	111	222	−127	138	231	82.99
LLDPE/5%QF/HDPE-g-MA	−124	143	223	---	113	204	−128	137	213	---
LLDPE/15%QF	−126	145	207	83.16	112	190	−127	138	197	79.10
LLDPE/15%QF/HDPE-g-MA	−125	143	197	---	112	179	−129	136	186	---
LLDPE/35%QF	−125	142	156	81.91	112	166	−128	137	148	77.71
LLDPE/35%QF/HDPE-g-MA	−124	143	151	---	111	144	−127	138	140	---

**Table 3:** Thermogravimetric analysis data for LLDPE/QF materials.

Sample	$T_{1\%}$ (°C)	$T_{5\%}$ (°C)	$T_{10}\%$ (°C)	$T_{50}\%$ (°C)	$T_{\max}\%$ (°C)	***RM*** (%)
Pure LLDPE	285	327	343	390	378; 410; 450; 501	1.12
LLDPE/5%QF	289	332	349	392	372; 456; 512	4.85
LLDPE/5%QF/HDPE-g-MA	282	333	352	394	377; 505	5.05
LLDPE/15%QF	299	348	369	414	396; 436; 509	13.53
LLDPE/15%QF/HDPE-g-MA	294	345	361	404	382; 455; 504	14.20
LLDPE/35%QF	301	358	384	433	409; 450; 508	27.40
LLDPE/35%QF/HDPE-g-MA	295	343	366	434	396; 439; 461; 507	26.62

**Table 4:** Vicat softening temperature (VST).

Sample	VST (°C)	Sample	VST (°C)
Pure LLDPE	126.36 ± 0.4	Pure LLDPE	126.36 ± 0.4
LLDPE/5%QF	123.86 ± 0.9	LLDPE/5%QF/HDPE-g-MA	125.91 ± 0.5
LLDPE/10%QF	**125.86 ± 0.3**	LLDPE/10%QF/HDPE-g-MA	**127.05 ± 0.6**
LLDPE/15%QF	124.03 ± 0.8	LLDPE/15%QF/HDPE-g-MA	124.15 ± 0.9
LLDPE/20%QF	**122.91 ± 0.3**	LLDPE/20%QF/HDPE-g-MA	**123.95 ± 1.1**
LLDPE/25%QF	123.61 ± 1.2	LLDPE/25%QF/HDPE-g-MA	**123.95 ± 1.2**
LLDPE/35%QF	123.53 ± 0.5	LLDPE/35%QF/HDPE-g-MA	125.35 ± 0.4

**Table 5:** Tensile strength (*σ_m_*) and relative elongation at maximum tensile stress (*ε_m_*) of LLDPE/QF and LLDPE/QF/HDPE-g-MA.

Sample	*σ_m_* (MPa)	*ε_m_* (%)	Sample	*σ_m_* (MPa)	*ε_m_* (%)
Pure LLDPE	24 ± 0.69	6 ± 0.36	Pure LLDPE	24 ± 0.69	6 ± 0.36
LLDPE/5%QF	**20.9 ± 0.82**	**4.9 ± 0.90**	LLDPE/5%QF/HDPE-g-MA	**22.7 ± 0.20**	**5.5 ± 0.99**
LLDPE/10%QF	17.4 ± 0.25	3.4 ± 0.29	LLDPE/10%QF/HDPE-g-MA	18.4 ± 0.75	3.6 ± 0.49
LLDPE/15%QF	11.2 ± 0.59	2.2 ± 0.32	LLDPE/15%QF/HDPE-g-MA	14.1 ± 0.77	2.2 ± 0.26
LLDPE/20%QF	10.6 ± 0.56	2.1 ± 0.33	LLDPE/20%QF/HDPE-g-MA	13.1 ± 0.58	2.1 ± 0.17
LLDPE/25%QF	10.3 ± 0.35	2.1 ± 0.46	LLDPE/25%QF/HDPE-g-MA	11.7 ± 0.85	2.0 ± 0.20
LLDPE/35%QF	**7.9 ± 0.79**	**1.2 ± 0.40**	LLDPE/35%QF/HDPE-g-MA	**11.4 ± 0.81**	**2.8 ± 0.21**

**Table 6:** Surface roughness parameters of the tested thin-walled moulded parts.

Sample	Surface Roughness Parameters. µm				
	*Ra*	*Rz*	*Rmax*	*Rq*	*RSm*
Pure LLDPE	1.06 ± 0.09	8.08 ± 0.02	4.62 ± 0.94	1.27 ± 0.12	0.60 ± 0.17
LLDPE/5%QF	**3.29 ± 0.75**	**17.36 ± 0.98**	**55.85 ± 1.55**	**4.64 ± 0.79**	0.66 ± 0.05
LLDPE/10%QF	4.80 ± 1.02	18.80 ± 1.08	33.53 ± 3.83	5.31 ± 0.42	**0.48 ± 0.07**
LLDPE/15%QF	6.34 ± 0.31	26.68 ± 1.39	70.10 ± 3.23	8.27 ± 1.02	0.63 ± 0.09
LLDPE/20%QF	5.42 ± 0.98	31.88 ± 2.84	71.08 ± 1.90	8.28 ± 0.89	0.59 ± 0.04
LLDPE/25%QF	6.30 ± 0.87	30.44 ± 1.56	75.60 ± 2.00	8.25 ± 1.36	0.70 ± 0.01
LLDPE/35%QF	**18.03 ± 1.21**	**69.07 ± 3.36**	**119.16 ± 4.54**	**22.23 ± 1.55**	**0.71 ± 0.05**
LLDPE/5%QF/HDPE-g-MA	**1.72 ± 0.25**	**10.03 ± 0.58**	**30.69 ± 1.33**	**2.58 ± 0.71**	**0.54 ± 0.05**
LLDPE/10%QF/HDPE-g-MA	2.36 ± 0.68	10.98 ± 1.04	38.20 ± 2.09	3.19 ± 0.72	0.70 ± 0.07
LLDPE/15%QF/HDPE-g-MA	6.39 ± 0.47	25.26 ± 2.26	63.54 ± 3.27	8.24 ± 0.87	0.64 ± 0.03
LLDPE/20%QF/HDPE-g-MA	8.31 ± 0.51	36.92 ± 1.29	89.10 ± 3.07	10.71 ± 1.07	0.77 ± 0.09
LLDPE/25%QF/HDPE-g-MA	7.30 ± 0.81	35.50 ± 4.01	106.05 ± 2.03	9.93 ± 0.27	0.78 ± 0.04
LLDPE/35%QF/HDPE-g-MA	**11.83 ± 0.23**	**56.88 ± 2.79**	**107.90 ± 5.21**	**15.87 ± 1.32**	**0.85 ± 0.04**

## Data Availability

The data presented in this study are available on request from the corresponding author.
